# Mesenchymal stem cells and their derived small extracellular vesicles for COVID-19 treatment

**DOI:** 10.1186/s13287-022-03034-4

**Published:** 2022-08-12

**Authors:** Yuling Huang, Xin Li, Lina Yang

**Affiliations:** 1 Departments of Geriatrics, The First Affiliated Hospital of China Medical University, Shenyang, 110001 Liaoning People’s Republic of China; 2 Departments of Infectious Disease, The First Affiliated Hospital of China Medical University, Shenyang, 110001 Liaoning People’s Republic of China

**Keywords:** Mesenchymal stem cells, Small extracellular vesicles, COVID-19, Molecular mechanism

## Abstract

Since December 2019, the coronavirus (COVID-19) pandemic has imposed huge burdens to the whole world, seriously affecting global economic growth, and threatening people’s lives and health. At present, some therapeutic regimens are available for treatment of COVID-19 pneumonia, including antiviral therapy, immunity therapy, anticoagulant therapy, and others. Among them, injection of mesenchymal stem cells (MSCs) is currently a promising therapy. The preclinical studies and clinical trials using MSCs and small extracellular vesicles derived from MSCs (MSC-sEVs) in treating COVID-19 were summarized. Then, the molecular mechanism, feasibility, and safety of treating COVID-19 with MSCs and MSC-sEVs were also discussed.

## Introduction

Coronavirus disease 2019 (COVID-19) has become an important issue that threatens human health worldwide. Since 2019, more than 2.6 × 10^8^ people have been diagnosed as having COVID-19 and more than 5 × 10^6^ died of the disease according to the latest statistics from the World Health Organization (WHO) (https://covid19.who.int/). The severe acute respiratory syndrome coronavirus 2 (SARS-CoV-2) is the direct cause of COVID-19, and it is transmittable by means of direct transmission, aerosol transmission, and contact transmission. Severe or fatal COVID-19 patients mainly show pathological features including diffuse alveolar damage, and changes related to coagulation disorders and/or hemodynamic depression [1]. Apart from affecting lung, SARS-CoV-2 can also infect pancreas, kidney, heart, nerve, and so on [2, 3]. Symptoms of COVID-19 range from asymptomatic infections to inducing critically ill symptoms including fever, headache, dry cough, diarrhea, hypoxemia, joint aches, metabolic acidosis, ARDS, multiple organ dysfunction, etc. [4]. While vaccination can prevent COVID-19 to some extent, the constantly emerging SARS-CoV-2 variants and mutations seem to reduce the protection afforded by vaccines [5]. In that context, timeous diagnosis and treatment have become a key to improving prognosis of COVID-19 patients [6]. Currently, therapeutic measures available for treatment of COVID-19 include respiratory support and drug therapy, such as plasma therapy, antiviral drug therapy, immune-mediated therapy, glucocorticoid therapy, metabolic support and nutrition therapy, stem cell therapy, artificial liver therapy, lung transplantation. [7]. Hence, developing new and effective treatment methods has become a focus among numerous researchers.

Transplantation of mesenchymal stem cells (MSCs) is a promising therapeutic strategy for a variety of disorders. Previous research has proven that MSCs are beneficial to treatment of many diseases. The International Society for Cellular Therapy (ISCT) defines MSCs according to the following three standards: plastic adhesion, specific surface markers, and trilineage differentiation [8]. MSCs are derived from bone marrow (BMMSCs), adipose tissue (ADMSCs), human umbilical cord Wharton’s-jelly (UC-MSCs or WJ-MSCs), etc*.* Many studies indicated that MSCs play their roles by differentiation and releasing various mediators, such as all kinds of soluble trophic factors and extracellular vesicles (EVs) to modulate immunity, anti-inflammatory, anti-apoptotic, and anti-viral [9, 10], which are the main mechanisms of current regimes to combat COVID-19. From basic to clinics, MSCs have been widely verified the potential to alleviate ARDS which plays an important role in COVID-19 [11, 12].

Compared to MSCs, EVs have a greater therapeutic potential due to nanoparticles that can penetrate the blood–brain barrier and reach injury locations, as well as lesser immunogenicity and tumorigenicity. EVs can be divided into small EVs (sEVs) with size of < 200 nm and medium/large EVs (m/lEVs) with size of > 200 nm in the Minimal Information for Studies of Extracellular Vesicles 2018 [13]. Functioning as a transporter, sEVs carry proteins, deoxyribonucleic acids (DNAs), messenger ribose nucleic acids (mRNAs), micro-ribose nucleic acids (miRNAs), etc.*.* specifically secreted by metrocyte [14]. sEVs wrapped by lipid bilayers can stably deliver those aforementioned key messages [15]. This explains why research on sEVs focuses on three aspects: use in treatment, serving as a drug carrier, and functioning as a biomarker. Santos and Almeida proposed that sEVs can be used as a vaccine against COVID-19 [16]. Fu and Xiong found that the engineered sEV system that is used for targeted delivery of potential antiviral drugs to specific tissues in vivo has therapeutic potential for SARS-CoV-2 infections [17]. Krishnamachary *e*t al. predicted the severity of COVID-19 disease using sEVs and lEVs [18]. Currently, many preclinical studies and clinical trials of MSCs and sEVs in treating COVID-19 are under investigation. Also, the molecular mechanism, feasibility, and safety of treating COVID-19 with MSCs and small extracellular vesicles derived from MSCs (MSC-sEVs) were discussed in this review.

## Current research into the use of MSCs against COVID-19

At present, several early‐phase clinical studies indicated that MSCs can combat COVID-19 by inhibition of cytokine storm, anti-inflammatory action, and immunoregulation. MSCs used in clinical trials are mainly UC-MSCs, and there are also MSCs derived from menstrual blood (MenSCs) or BMMSCs. Many clinical trials have validated the feasibility, safety, and tolerance of MSCs and MSC-sEVs in treating COVID-10 (Table 1).

**Table 1:** Clinical trials of MSCs and MSC-sEVs against COVID-19

Study Phase and Type	Severity of COVID-19	Number Enrolled	MSC/MSC-sEV source	Dosage	Frequency	Assessment of the efficacy	Adverse Primary Safety Outcome	Refs.
A case report	Critical	1	UC-MSCs	1 × 10^6^ cells/kg	Days 0 and 3	Inflammation-related indicators significantly improved; the cytokine storm was dampened and the NK cells were modulated	No infusion or allergic reactions, secondary infections, or treatment-related adverse events were found	[21]
A case report	Severe	1	WJ-MSCs	1 × 10^6^ cells/kg	Single dose	The pulmonary function and symptoms were significantly improved	No acute infusion-related or allergic reactions were observed	[22]
A case report	Critical	1	UC-MSCs	1 × 10^6^ cells/kg	Single dose	Inflammatory reaction was improved, and lung function and multiple organ functions were improved	No obvious side effects were observed	[23]
A small sample, single arm, pilot trial	Severe 9; critical 7	16	UC-MSCs	1 × 10^8^ cells	Single dose	The oxygenation index was improved, mortality relatively lowered; radiological presentations improved, lymphocyte count recovered and cytokine levels decreased	No infusion-related or allergic reaction	[24]
A Phase 1 parallel non-randomized assigned, controlled, trial	Moderate 9; severe 9	18	UC-MSCs	3 × 10^7^ cells	Days 0, 3, and 6	The levels of cytokine reduced; symptoms improved	No serious UC-MSC infusion-associated adverse events were observed	[25]
A Phase 2 randomized, double-blind, placebo-controlled trial	Severe	101	UC-MSCs	4 × 10^7^ cells	Days 0, 3, and 6	Accelerated resolution of lung solid component lesions and the integrated reserve capability improved	No MSC infusion-related adverse events	[26]
A prospective cohort follow-up study	Severe	28	UC-MSCs	2 × 10^6^ cells/kg	Single dose	Accelerated partial pulmonary function recovery and improved HRQL	No obvious adverse effects were observed in the UC-MSC group after 3 months	[27]
A Phase 1/2 trial	Severe 111; critical 99	210	UC-MSCs	1–2 × 10^6^ cells/kg	Single dose	The SaO2 parameter tended to improve; significantly higher survival was observed in patients who underwent UC-MSCs	No adverse effects were observed related to infusion or allergic reactions, secondary infection, or life-threatening adverse events	[28]
A Phase 1 double-blind, multi-center, randomized controlled trial	Critical	40	UC-MSCs	1 × 10^6^ cells/kg	Single dose	The survival rate increased; there was no significant difference regarding the period of intubation and the period from intubation	MSCs were well tolerated with no life-threatening complications or acute allergic reactions during the administration	[29]
A Phase 1/2a double-blind randomized controlled trial	Mild-to-moderate 6; moderate-to-severe 18	24	UC-MSCs	100 ± 20 × 10^6^ cells	Days 0 and 3	The levels of key inflammatory molecules were reduced; time to recovery was significantly shorted	No serious adverse events related to MSC infusion were observed	[30]
Phase 1	Severe	5	WJ-MSCs	150 × 10^6^cells	Days 0, 3, and 6	Inflammation was reduced; COVID-19 antibody tests rose the total score of zonal involvement in both lungs was improved	No serious complications were observed except the headache in one of them	[31]
A case series	Critical	11	PL-MSCs, UC-MSCs	200 × 10^6^ cells	Days 0, 2, and 4	Respiratory symptoms improved and inflammatory conditions reduced	No serious adverse events were reported 24–48 h after the cell infusions	[32]
A non-randomized assigned, controlled trial	Severe	23	BMMSCs	1 × 10^6^ cells/kg	2–3 times	Pulmonary function and overall outcome improved	No significant side effects after MSC infusion	[33]
2 case reports	Severe	2	MenSCs	1 × 10^6^ cells/kg	Days 0, 1, and 3	Lung function improved	Not find obvious adverse reactions	[34]
A Phase 1 multi-center, open-label, non-randomized, parallel, controlled trial	Severe 26; critical 18	44	MenSCs	9 × 10^7^ cells	Days 0, 2, and 4	The mortality significantly lowered; alleviating the breathing difficulties and reducing the symptoms of ARDS or expiratory dyspnea	The incidence of most adverse events did not differ between the groups, experimental group, and control group	[35]
A case	Severe	1	UC-MSCs	1.1 × 10^6^ cells/kg	Days 0, 2, and 8	Inflammatory, respiratory, thrombotic, and renal parameters improved	No adverse events occurred	[41]
A prospective double phase 1/2 controlled trial	Moderate 10; critical 20	30	WJ-MSCs	3 × 10^6^ cells/kg	Days 0, 3, and 6	All the indicators of anti-inflammation, antifibrosis signs in the lungs, and immune-modulatory markers improved	No adverse or serious adverse events occurred related to the MSC therapy	[42]
A case series	Severe 23; critical 8	31	UC-MSCs	1 × 10^6^ cells/kg	1–3 times	SARS-CoV-2 PCR results of 30 patients (96·8%) became negative after a mean time of 10·7 days; laboratory parameters, hypoxia, immune reconstitution, and cytokine storms improved	No adverse events were attributable to intravenous transplantation of UC-MSCs	[43]
A Phase 1/2a randomized controlled trial	Severe	24	UC-MSCs		1–3 times	Survival, serious adverse events-free survival, and time to recovery significantly improved	Serious adverse events-free	[44]
A Phase 1, single-arm, non-randomized, parallel trial	Healthy	24	ADMSC-sEVs	2–16 × 10^8^ particles	Once inhalation	Improved survival rate to 80% at 96 h in P. aeruginosa-induced murine lung injury model by decreasing lung inflammation and histological severity	No serious adverse events were observed within 7 days	[78]
A prospective nonblinded non-randomized trial	Mild 1; severe 20; critical 3	24	BMMSC-sEVs	15 mL	Single dose	Patients’ clinical status and oxygenation improved, laboratory values revealed significant improvements in absolute neutrophil count, and acute phase reactants declined	No adverse events were observed within 72 h of ExoFlo administration	[79]

Many clinical trials have validated the feasibility, safety, and tolerance of MSCs in treating COVID-10. MSCs used in clinical trials are mainly UC-MSCs or WJ-MSCs, and there are also MenSCs or BMMSCs. As for the dose of intravenous infusion of MSCs, the majority of studies adopt three doses, each with 106 cells/kg in 100 mL of normal saline

MSCs: mesenchymal stem cells; UC-MSCs or WJ-MSCs: MSCs are derived from human umbilical cord Wharton’s-jelly; BMMSCs: MSCs are derived from bone marrow; MenSCs: MSCs derived from menstrual blood; MSC-sEVs: small extracellular vesicles derived from MSCs

### Clinical trials of MSCs against COVID-19

Currently, more and more clinical trials are underway to study the use of MSCs in treating COVID-19. After being transplanted to COVID-19 patients, whether MSCs will be infected by SARSCoV-2 or maintain their therapeutic effect is the premise of using MSCs. Schäfer et al. unveiled that MSCs are tolerant to SARS-CoV-2 infections and can maintain their immunoregulatory potential, which supports their potential applicability in the treatment of COVID-19 [19]. Through evaluation, Wedzinska et al. found the possible changes in biology of MSCs in an active inflammatory environment: no matter what the aerobic condition is, the external inflammatory environment will not induce phenotypic changes in WJ-MSCs or cause disruption of proliferation; it will also not inhibit the secreting characteristics of these MSCs, so that they can be used to fight acute inflammation [20].

MSCs have been recognized as safe and feasible in treatment of patients with COVID-19 pneumonia and many clinical trials confirmed that the intravenous injection of MSCs will relieve clinical features and not induce severe adverse effects. It has been reported that a severe SARS-CoV-2 infected patient separately diagnosed in Changsha (Hunan Province) [21] and Liaocheng (Shandong Province) [22], received MSC infusion with inflammation-related indicators and the pulmonary function was significantly improved, while no adverse reaction associated with MSC treatment occurred. In addition, after applying UC-MSC infusion as an adjuvant therapy for a critically severe COVID-19 patient, Zhu et al. found that the absolute number of lymphocytes increased significantly and even multiple organ functions were ameliorated while no obvious side effect occurs [23]. Feng et al. conducted four rounds of transplantation of UC-MSCs to 16 severe and critically severe COVID-10 patients and recorded adverse events from registration to the 28^th^ day of treatment. Results showed that the oxygenation index was enhanced and mortality relatively lowered, however, there was no infusion-related adverse events or anaphylaxis [24]. Meng et al. conducted a parallel, controlled non-randomized phase 1 clinical trial, to evaluate the safety of infusion of UC-MSCs in treating moderate and severe COVID-19 patients with pulmonary diseases, and observed that the levels of cytokine reduced, symptoms improved, and no severe adverse events related to the infusion [25]. Furthermore, Shi et al. recruited 101 severe COVID-19 patients with lung injury to receive UC-MSCs.The lung solid component lesions resoluted faster and the integrated reserve capability improved. All adverse events in the observation period of the randomized double-blinded placebo-controlled phase 2 trial (NCT04288102) are unrelated to the UC-MSC intervention [26]. Different from the short-term observation of adverse reactions, Feng et al. conducted a three-month post-discharge follow-up. They found that vein transplantation of UC-MSCs facilitates recovery of some lung function and improves the health-related quality of life, while no patients show adverse reactions after discharge [27]. As for the dose of intravenous infusion of MSCs, there remains a lack of a unified standard. The majority of studies adopt three doses, each with 10^6^ cells/kg in 100 mL of normal saline. There are also clinical trials using single and two injections, which may be dependent on the severity of patients. N et al. performed UC-MSC treatment on 210 severe and critically severe patients through intravenous injection of single-dose clinical-grade UC-MSCs (1 × 10^6^ to 2 × 10^6^ cells/kg). They found that repeated doses can be applied for seriously ill patients, to support the treatment primarily through the anti-inflammatory and immunoregulatory effects [28]. In a randomized controlled trial, 20 patients received single intravenous infusion of UC-MSCs (1 × 10^6^ cells/kg). The significant decreases in interleukin 6 and the reducing number of circulating peripheral blood immune cells suggest the recession of the cytokine storm and progress in clinical improvement [29]. In a double-blinded 1/2a phase randomized controlled trial, the subjects in the UC-MSCs treated group received two intravenous infusions (100 ± 20 × 10^6^ cells) and no serious adverse events associated with the infusion of UC-MSCs were observed [30]. Five severe COVID-19 patients received three intravenous injections of WJ-MSCs (each 150 × 10^6^ cells) every three days and no severe complications related to WJ-MCSs were found [31]. In a phase 1 clinical trial, Hashemian et al. demonstrated the safety, feasibility, and tolerance of multiple high-dose MSCs from allogenic placenta and UC-MSCs in treating critically severe acute respiratory distress syndrome (ARDS) patients induced by COVID-19 [32].

Almost all of the aforementioned research uses UC-MSCs to treat COVID-19. This is possibly because UC-MSCs are easily isolated and cultured, have strong proliferation ability, and are derived from a relatively pure source with less chance of contamination. BMMSCs and MenSCs have also been used to combat SARS-CoV-2 in clinical trials, in addition to UC-MSCs. Häberle et al. treated five of 23 severe COVID-19 patients with ARDS by infusion of BMMSCs. In this way, the lung functions and overall prognosis of the patient population are also improved [33]. Tang et al. revealed that allogeneic MenSCs also improve lung function through the anti-inflammatory effect on injured lung [34]. Results of a multicenter open-label non-randomized parallel-controlled phase I clinical trial show that transplantation of MenSCs can significantly reduce the mortality of COVID-19 induced by severe SARS-CoV-2 infections [35]. It is noteworthy that a preclinical trial reveals that human dental pulp stem cells can regulate generation of cytokines in COVID-29 patients through in vitro peripheral blood mononuclear cells [36].

To treat SARS-CoV-2 infections, the combination of injection of MSCs and other therapies seems to have better treatment effects. Peng et al. confirmed that UC-MSCs and serum of convalescent patients have collaborative features in inhibiting cytokine storm, facilitating repair of lung injury, and recovering lung function [37]. Senegaglia et al. reported treatment of severe COVID-19 patients with the combination of monoclonal antibody tocitizumab and UC-MSCs, with no adverse reaction [38]. Commonly seen complications of COVID-19 patients include thrombotic complication [39]. Intravenous injection of MSCs is also likely to induce blood coagulation events, while O’Rourke et al. found that intravascular thrombosis is inhibited by delivery of MSCs using a preclinical trial system involving an in vitro bioreactor [40].

After being exposed to external stimulation, numerous cytokines are released, which induces hyperimmunity of the organism and finally affects various systems. Inhibitions of cytokine storm and anti-inflammation are the main mechanisms of action of MSCs against COVID-19. Ciccocioppo et al. conducted immunological research on a patient hospitalized due to COVID-19 pneumonia and treated with UC-MSCs: they found that UC-MSCs may be beneficial to developing an anti-inflammatory and protective environment, which can inhibit the cytokine storm and help recovery of the pulmonary alveolar capillary barrier, instead of having a direct antiviral effect [41]. Adas et al. proved that, in addition to reducing mortality and shortening the length of stay in ICU, WJ-MSCs also play a special role in treatment of patients with critically severe COVID-19: MSC transplantation seems to control the cytokine storm and reduce the progression of the disease [42]. Guo et al. suggested that treatment with UC-MSCs can recover oxygenation in hospitalized severe COVID-19 patients and down-regulate the cytokine storm while not inducing any response to infusion [43]. In a double-blinded phase 1/2a randomized controlled trial (*n* = 24), Kouroupis et al. determined the plasma concentrations of sTNFR2, TNFα, and TNFβ. Their results indicated that sTNFR2 exerts an inflammation inhibiting effect when mediating influences of UC-MSCs on the plasma concentrations of TNFα and TNFβ [44].

### Possible molecular mechanisms of MSCs against COVID-19

**Fig. 1:**
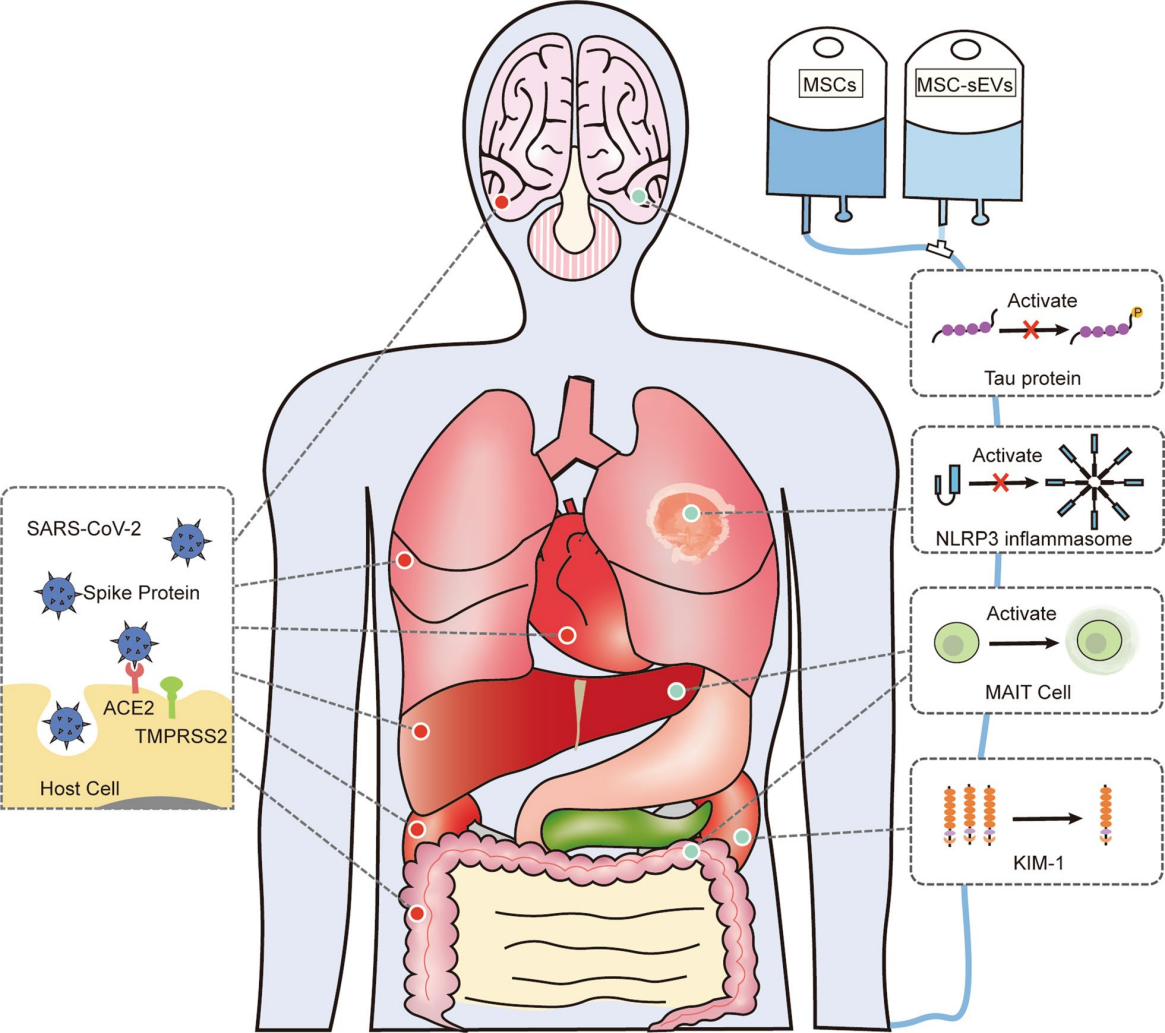
Possible molecular mechanisms of MSCs against COVID-19. ACE2 and TMPRSS2 are common pathways for SARS-CoV-2 to infect each organ. SARS-CoV-2 enters target cells via its S protein binding ACE2. Meanwhile, SARS-CoV-2 can damage various organs by virtue of specific factors, such as Tau protein in brain, KIM-1, and NLRP3 inflammasome, and MAIT cells. MSCs, mesenchymal stem cells; ACE2, angiotensin converting enzyme 2; TMPRSS2, transmembrane serine proteinase 2; KIM-1, kidney injury molecule-1; MAIT cells, mucosal-associated invariant T cells

After being infected with SARS-CoV-2, the body develops diverse immune responses and inflammation to combat COVID-19 [45, 46]. ACE2 and TMPRSS2 are common pathways for SARS-CoV-2 to infect many organs [47]. Meanwhile, SARS-CoV-2 can damage various organs by virtue of specific factors, such as Tau protein in brain [48], kidney injury molecule-1 (KIM-1) [49], and NLRP3 inflammasome [50]. A coincidence arises whereby numerous studies confirmed that MSCs can influence these common and specific factors, thus protecting corresponding organs. Therefore, MSCs may be able to combat COVID-19 by altering these processes (Fig. 1).

#### Common: ACE2 and TMPRSS2

SARS-CoV-2 enters cells through interactions of its spike (S) protein with angiotensin converting enzyme 2 (ACE2) receptor on cells. SARS-CoV-2 is internalized with the aid of transmembrane serine proteinase 2 (TMPRSS2) [47]. ACE2 and TMPRSS2 are distributed extensively across various organs, including the lung [51], small intestine [52, 53], heart [54], and brain [55], and kidney [56], so that SARS-CoV-2 can affect various organs [57]. The low expressions of ACE2 and TMPRSS2 in MSCs provide a premise for treating COVID-19. Leng et al. found that MSCs are ACE2(-) and TMPRSS2(-) through gene expression profiling, which implies that MSCs are not infected by SARS-CoV-2 [58]. By evaluating MSCs derived from amniotic membrane, umbilical cord blood, UC-MSCs, ADMSCs, and BMMSCs, Avanzini et al. found that ACE2 and TMPRSS2 are expressed in Calu-3 cell strains in lung, rather than in all MSCs [59]. Hernandez et al. confirmed the negative expression of ACE2 and low expression of TMPRSS2 in 24 batches of UC-MSCs [60]. Furthermore, Desterke et al. proposed that early cultured UC-MSCs express even lower ACE2 [61]. Intrestingly, Wei et al. proposed that ACE2 overexpressing MSCs can moderate COVID-19 lung injury in vivo and in vitro by decreasing inflammatory factors and pyroptosis factors [62].

#### Specific: Tau protein, KIM-1, NLRP3 inflammasomes, and mucosal-associated invariant T cells

SARS-CoV-2 can damage the body through some specific mechanisms (Tau protein in brain, KIM-1 in kidney, and NLRP3 inflammasomes), in addition to the common ones (ACE2 and TMPRSS2). Overall, MSCs and sEVs protect the body through the above mechanisms to against COVID-19 (Table 2).

**Table 2:** Possible molecular mechanisms of MSCs against COVID-19

	Molecular	Mechanisms of SARS-CoV-2	Refs	Mechanisms of MSCs	Involved organs/diseases	Involved cell types	Origin of MSCs	Refs.
Common molecular	ACE2 and TMPRSS2	SARS-CoV-2 enters target cells via its S protein which helps the virus to target ACE2 binding sites of cells, and the priming activator TMPRSS2 of ACE2 assists internalization of the virus	[51]	ACE2(-) and TMPRSS2(-) protect MSCs from infecting by SARS-CoV-2	Lung	Immune cells	BMMSCs, ADMSCs, UC-MSCs, etc	[58–61]
				ACE2 overexpressing MSCs decrease inflammatory factors and pyroptosis factors	Lung	AT-II and Beas-2B	UC-MSCs	[62]
Specific molecular	Tau protein	S protein of SARS-CoV-2 interacts with Tau protein	[64]	Regulate hyperphosphorylated Tau protein	Brain	Nerve cells	UC-MSCs	[65–67]
	KIM-1	KIM-1, a potential receptor of SARS-CoV-2, mediates and exacerbates the vicious circle of kidney infections by the virus	[49]	Reduce the KIM-1 level	Kidney		BMMSCs	[70]
	NLRP3 inflammasomes	The interplay between ACE2 receptor and SARS-CoV-2 S protein activates NLRP3 inflammasomes, thus facilitating inflammatory responses	[50]	Inhibit activation of inflammatory mediators and NLRP3 inflammasomes via exosomes	Intervertebral disc	Nucleus pulposus cells	BMMSCs	[71]
				Block the NLRP3 inflammasome activation and inflammatory agents	Type 2 diabetes		UC-MSCs	[72]
				Control NLRP3 by facilitating the Hippo pathway of macrophages and regulating XBP1	Liver	Macrophage	BMMSCs	[73]
				Inhibit NLRP3 inflammasome activity via the anti-oxidative protein stanniocalcin-1	Heart	HL-1 cells	BMMSCs	[74]
	MAIT cells	SARS-CoV–2 activates and depletes MAIT cells that can kill bacteria or cells infected by viruses	[75]	Induce activated phenotypes and regulate activation of MAIT cells by up-regulating expressions of CD69, granzyme B, IFN-γ, and TNF-α	Infection, metabolic disorders, and inflammatory diseases	MAIT cells	ADMSCs	[76]

SARS-CoV-2 can damage the body through some specific mechanisms (Tau protein in brain, KIM-1 in kidney, and NLRP3 inflammasomes), in addition to the common ones (ACE2 and TMPRSS2). Overall, MSCs and sEVs protect the body through the above mechanisms to against COVID-19

ACE2: angiotensin converting enzyme 2; TMPRSS2: transmembrane serine proteinase 2; KIM-1: kidney injury molecule-1; MAIT cells: mucosal-associated invariant T; MSCs: mesenchymal stem cells; BMMSCs: MSCs are derived from bone marrow; UC-MSCs or WJ-MSCs: ADMSCs: MSCs derived from adipose tissue

SARS-CoV-2 can enter the central nervous system through olfactory mucosa [63] The hyperphosphorylation of Tau protein is one of the features of neuroinvasion of SARS-CoV-2. The S protein of SARS-CoV-2 interacts with amyloid proteins, such as Ab, a-synuclein, Tau, prion, and TDP-43 RRM [64], which lead to changes in distribution of Tau from axon to somatic cells, hyperphosphorylation, and apparent neuronal death [48]. By coincidence, Jia et al. repaired damaged nerve cells by down-regulating hyperphosphorylated Tau protein, reversing spinal loss, and facilitating synaptic plasticity using UC-MSCs [65]. Other researchers also reduced the hyperphosphorylation of Tau protein in the mouse model of Alzheimer’s disease by intravenous administration of MSCs [66, 67].

A retrospective analysis indicated that SARS-CoV-2 directly infects kidney and mediates renal tubular acidosis and AKI [68]. Tanase et al. identified KIM-1 as a potential marker of kidney injury [69]. Yang et al. believed that KIM-1 is a potential receptor of SARS-CoV-2, and it mediates and exacerbates the vicious circle of kidney infections by SARS-CoV-2 [49]. Coincidentally, Aussel et al. reduced the KIM-1 level on kidney slices by infusion of MSCs [70].

SARS-CoV-2 can induce inflammation in many ways. Ratajczak et al. found that the interplay between ACE2 receptor and SARS-CoV-2 S protein in human vascular and hematopoietic stem cells activates NLRP3 inflammasomes, thus facilitating inflammatory responses. If NLRP3 inflammasomes are activated excessively, pyroptosis may be triggered [50]. MSCs were found to inhibit the activation of NLRP3 inflammasomes in many diseases. In degeneration of intervertebral discs, Xia et al. reported that MSCs play an anti-inflammatory role by inhibiting activation of inflammatory mediators and NLRP3 inflammasomes via exosomes [71]. Sun et al. proved that UC-MSCs enhanced insulin resistance by suppressing inflammation in rats with type-2 diabetes mediated by NLRP3 inflammasomes [72]. Research results of Li et al. indicated that MSCs control the activation of NLRP3 by facilitating the Hippo pathway of macrophages and regulating XBP1 [73]. Miteva et al. also confirmed that MSCs limit the adverse outcome of cardiac and systemic NLRP3 inflammasome activation in Coxsackievirus B3-induced myocarditis [74].

T cells are one of the core immune cells in the defense against SARS-CoV-2. Mucosal-associated invariant T (MAIT) cells are immune cells in viral inflammation. Hubrack et al. showed that SARS-CoV–2 activates and depletes MAIT cells that can kill bacteria or cells infected by viruses. In COVID-19 patients, the granzyme B, IFN-γ, TNF-α, and perforin of MAIT cells were found in lowered proportions [75]. MSCs significantly induce activated phenotypes and then regulate activation of MAIT cells by up-regulating expressions of CD69, granzyme B, IFN-γ, and TNF-α but not by influencing proliferation of MAIT cells in bone marrow, liver, and intestinal tissue [76].

## MSC-sEVs Against COVID-19

The ISCT and the International Society for Extracellular Vesicles (ISEV) have recognized the potential of MSC-sEVs and sEVs from other cells in treatment of COVID-19. Research by Vaka et al. revealed that the paracrine production and viability of BMMSC, heart-derived cells, and UC-MSCs under COVID‑19 ARDS cytokines are not altered, laying the groundwork for MSC-sEVs to treat COVID‑19 [77].

At present, there are a few clinical research on the treatment of COVID-19 with MSC-sEVs (Table 2). Despite this, existing clinical trials all confirmed that MSC-sEVs are safe and effective. Shi et al. performed complex research to evaluate the preclinical efficacy and safety of clinical-grade aerosolized allograft ADMSC-sEVs. On the one hand, the aerosol inhalation of ADMSC-sEVs protects mice against severe pneumonia; on the other hand, it is safe for healthy volunteers and has no severe adverse effects (NCT04313647) [78]. However, the authors used human platelet lysate for sEVs preparation and PEG methods to isolate sEVs which is controversy. In hospitalized patients with severe COVID-19, single intravenous injection of MSC-sEVs significantly improves the hypoxia, immune reconstitution, and cytokine storm and there is no adverse reaction related to the infusion of sEVs [79]. The absence of blinding, randomization, and the limited sample size require further clinical studies to investigate safety and efficacy of sEVs treating COVID-19. The dosage, the methods of isolation and many other issues of above trials have been summarized in Table 1. Many ongoing clinical trials of MSC-sEV treatment in COVID-19 pneumonia and ARDS (NCT04493242, NCT05354141) may provide evidence to support MSC-sEV as a cell-free therapy for COVID-19.

According to some preclinical research, MSC-sEVs appear to alleviate COVID-19 via antiviral (preventing viral duplication and transmission) and anti-inflammatory effects. Chutipongtanate et al. suggested that MSC-sEVs exhibit anti-SARS-CoV-2 effects by inducing infected lung epithelial cells to decrease viral replication and production/release of infectious virions [80]. Park et al. proved that MSC-sEVs and five main miRNAs (miR-92a-3p, miR-26a-5p, miR-23a-3p, miR-103a-3p, and miR-181a-5p) significantly inhibit duplication of SARS-CoV-2 and have anti-inflammatory activity in vitro [81]. In addition to the antiviral effect, MSC-sEVs control inflammation to treat COVID-19. Khanh et al. proved that WJ-sEVs have the potential to decrease the cytokine storm in patients with chronic inflammation and viral infections by in vitro experiments [82]. In the Silico analysis, Schultz et al. proposed that miRNAs carried by MSC-EVs (including MSC-sEVs) serve as potential multiple targets for treatment of COVID-19, which reduces the excessive generation of inflammatory factor and chemokines, blood coagulation cascade, and apoptosis [83]. Complement and neutrophil axis can amplify and perpetuate the cytokine storm in severe COVID-19 patients. Recent research determine that MSC-sEVs can specifically inhibit they through CD59[84].

## Conclusions

In summary, SARS-CoV-2 can infect the body through ACE2 and TMPRSS2, as well as Tau protein, KIM-1, NLRP3 inflammasomes, and MAIT cells. MSCs and MSC-sEVs show the possibility in treating COVID-19 via the molecular mechanism mentioned above. MSCs have been approved as a drug for clinical treatment of Crohn’s disease complicated by intestinal fistula and graft-versus-host disease. Numerous early‐phase studies have verified the feasibility, effectiveness, and safety of MSCs and MSC-sEVs, while their optimal sources and specific medication regimens still remain an open question.

From existing research, UC-MSCs are most widely used and they are mainly administered through continuous intravenous injection (1 × 10^6^ cells/kg). Meanwhile, improvement of the extraction, culture, and proliferation of MSCs can also enhance the treatment effect. For example, 3D cell culture strengthens the angiogenesis and immunoregulatory functions of MSCs [85]. Moreover, the acquisition of abundant and scalable MSC-sEVs and sEVs damage during extraction are also matters of concern. Lee et al. combined bioreactor culture with tangential flow filtration and size exclusion chromatography to produce highly-pure bioactive sEVs carrying hetIL-15/lactadherin [86], providing a clue for acquiring a large amount of MSC-sEVs. As for possible contamination, Good Manufacturing Practice (GMP) criteria and the critical quality control points (CQCP) may aid in evaluating the safety of clinical-grade MSCs and MSC-sEVs.

There are some other limitations to this topic. Firstly, in light of MSC conservation and transportation, it is critical to assess the differences in therapeutic properties between thawed and fresh MSCs. Secondly, preclinical and clinical study endpoints should include other laboratory metrics such as SARS-CoV-2 viral titers in addition to clinical features. Thirdly, most clinical trials have a short follow-up, which cannot observe the effect of MSCs and MSC-sEVs on long-term complications of COVID-19. The other issue is the administration of other drugs which may affect the anti-inflammation and immunomodulatory properties of MSCs and MSC-sEVs.

Taken together, more extensive randomized controlled research needs to be undertaken to reveal the detailed pathophysiological mechanism underlying the treatment of COVID-19 with MSCs and MSC-sEVs. Clinically, further phase 3 clinical trials should be conducted to ascertain the potential of MSCs and MSC-sEVs in treating COVID-19.

## Data Availability

Not applicable.

## Abbreviations

COVID-19: Coronavirus disease 2019
SARS-CoV-2: Severe acute respiratory syndrome coronavirus 2
MSCs: Mesenchymal stem cells
BMMSCs: MSCs derived from bone marrow
ADMSCs: MSCs derived from adipose tissue
UC-MSCs: MSCs derived from human umbilical cord
WJ-MSCs: MSCs derived from Wharton’s-jelly
EVs: Extracellular vesicles
sEVs: Small EVs
MSC-sEVs: Small extracellular vesicles derived from MSCs
S protein: Spike protein
ACE2: Angiotensin converting enzyme 2
TMPRSS2: Transmembrane serine proteinase 2
KIM-1: Kidney injury molecule-1
IFN: Interferon
AKI: Acute kidney injury
MAIT cells: Mucosal-associated invariant T cells
MenSCs: MSCs derived from menstrual blood
ARDS: Acute respiratory distress syndrome
